# Supplementary material for: Mapping the evolution of fertility support policies in China: A content and instrumental analysis
Source: PLoS One. 2025 Oct 9;20(10):e0332137. doi: 10.1371/journal.pone.0332137 (PMC12510515; doi:10.1371/journal.pone.0332137)
Supplement: S1 Appendix — (ZIP) [file pone.0332137.s001.zip › S1 Appendix. 226 original policy documents/66-国务院妇女儿童工作委员会关于印发《国务院妇女儿童工作委员会各成员单位职责》的通知(FBM-CLI-4-138216).doc]

国务院妇女儿童工作委员会关于印发《国务院妇女儿童工作委员会各成员单位职责》的通知

**发布部门：** [国务院妇女儿童工作委员会](https://www.pkulaw.com/law/chl?Aggs.IssueDepartment=7060250)

**发文字号：**国妇儿工委字〔2009〕3号

**发布日期：**2009.01.14

**实施日期：**2009.01.14

**时效性：** [现行有效](https://www.pkulaw.com/law/chl?Aggs.TimelinessDic=01)

**效力级别：** [部门工作文件](https://www.pkulaw.com/law/chl?Aggs.EffectivenessDic=XE0303)

**法规类别：** [老少妇幼残保护](https://www.pkulaw.com/law/chl?Aggs.Category=01604)

国务院妇女儿童工作委员会关于印发《国务院妇女儿童工作委员会各成员单位职责》的通知
（国妇儿工委字〔2009〕3号）

国务院妇女儿童工作委员会各成员单位：
　　国务院妇女儿童工作委员会于2008年12月30日召开了国务院妇女儿童工作委员会五届一次全体会议。现将国务院妇女儿童工作委员会全体会议讨论通过的《国务院妇女儿童工作委员会各成员单位职责》印发给你们，请遵照执行。

国务院妇女儿童工作委员会
二○○九年一月十四日

国务院妇女儿童工作委员会各成员单位职责
（2008年12月30日）

　　中宣部：协调和指导地方宣传机构、新闻媒体，通过大众化的信息手段，宣传男女平等基本国策和儿童优先原则，宣传《[中国妇女发展纲要](https://www.pkulaw.com/chl/37733dd7246afab4bdfb.html)》和《中国儿童发展纲要》，创造有利于妇女儿童发展的舆论环境；引导各种媒体传播有益于妇女发展和儿童成长的社会、文化信息。
　　外交部：积极向国际社会宣传我国在妇女儿童领域取得的成就，协助相关部门参与妇女儿童事务国际交流与合作。
　　国家发改委：将妇女儿童事业发展纳入国民经济和社会发展中长期规划和年度计划；在拟订并组织实施规划、制定政策中，注重协调经济社会发展与妇女儿童事业发展的关系，维护妇女儿童权益；加强妇女儿童事业建设，促进妇女儿童事业的持续发展。
　　教育部：保障所有儿童受教育权利；在各级各类学校招生及中等以上学校毕业生就业中坚持男女平等原则；提高家庭教育水平，发展特殊教育、成人继续教育、职业教育、实用技术培训，提高妇女的教育水平；尊重女教师，关心和保障女教职工的合法权益。
　　科技部：组织开展有关妇女儿童身心健康等方面的科学技术研究及其成果的推广应用工作；关心和保障女科技工作者合法权益，为发挥妇女在科技发展中的作用创造条件。
　　国家民委：宣传、推动少数民族和民族地区贯彻执行国家有关妇女儿童的法律、法规；组织研究少数民族妇女儿童保护等问题并提出有关政策措施；配合有关部门维护和保障少数民族妇女儿童的合法权益。
　　公安部：打击侵害妇女儿童人身权益和卖淫嫖娼等违法犯罪活动；牵头协调开展反对拐卖妇女儿童犯罪工作。
　　民政部：负责属于社会救助对象的妇女儿童的权益保护和生活救助的管理工作；做好孤儿、弃婴的收养、治疗、康复、教育工作和流浪儿童以及特殊困难的残疾儿童的救助保护工作；做好属于“三无对象”（无法定抚养人、无劳动能力、无生活来源）和孤老妇女的供养工作。
　　司法部：加强有关保护妇女儿童权益的法律、法规宣传；做好女性罪犯和劳动教养人员及未成年罪犯、未成年劳动教养人员、未成年强制隔离戒毒人员和未成年政府收容教养人员的教育改造、教育矫治工作；建立健全法律援助体系，为维护妇女儿童合法权益提供法律援助。
　　财政部：根据妇女儿童事业发展的需要和国家财力，提供必要的经费，并监督检查经费的使用情况。
　　人力资源社会保障部：促进各类妇女人才的成长和合理配置；在公务员考试录用、专业技术职务评聘、高校毕业生就业中，保障男女平等原则的落实。保障女性在就业、培训、劳动报酬以及技工学校招生等方面与男性平等的权利；保障女职工劳动保护法规、政策的落实；禁止使用童工；组织下岗失业女职工的职业技术培训，扩大妇女就业领域；逐步建立和完善生育保险制度。
　　环境保护部：通过开展绿色家庭、绿色学校等绿色创建活动及其他活动，加强环保知识、法律法规、环境道德等的宣传，提高广大妇女儿童的环境意识；支持和鼓励妇女儿童参加各种环境保护的宣传教育和其他有关部门活动，提高妇女参与环境保护及决策的程度，充分发挥妇女在环境保护中的作用。
　　住房城乡建设部：在拟订城乡规划、住房保障和市政公用事业的政策法规以及工程建设标准规范工作中，注意保障妇女儿童权益，创造适宜的居住环境。
　　水利部：指导农村饮水安全工作，保障农村居民饮水安全；加强水资源的节约、保护和合理配置，指导乡镇供水，保障城乡供水安全，不断改善村镇居民饮用水条件。
　　农业部：做好农村妇女的农业技术培训工作，努力提高农村妇女的劳动技能；支持和鼓励妇女兴办和参加生态农业建设、农村可再生能源综合开发利用建设等事业，充分发挥农村妇女在发展农业和农村经济中的作用。
　　商务部：积极争取国际援助，支持妇女儿童工作；积极争取联合国和其他国际组织及双边捐助国对我国妇女儿童事业发展，特别是对实施《[中国妇女发展纲要](https://www.pkulaw.com/chl/37733dd7246afab4bdfb.html)》、《中国儿童发展纲要》的项目支持，为扩大有关国际合作和国际交往创造条件。
　　文化部：丰富妇女儿童文化生活，推进妇女儿童文化艺术事业的发展；宣传妇女在两个文明建设中的作用、业绩和贡献，展现当代女性风貌。
　　卫生部：认真贯彻实施《[中华人民共和国母婴保健法](https://www.pkulaw.com/chl/ea633acd04298a40bdfb.html)》等卫生法律、法规，落实《[中国妇女发展纲要](https://www.pkulaw.com/chl/37733dd7246afab4bdfb.html)》和《中国儿童发展纲要》有关卫生指标；普及妇幼保健、生殖健康以及疾病预防等卫生科学知识；改善妇幼保健服务设施和条件，提高服务能力；依法进行妇幼保健、生殖健康服务和管理，降低孕产妇死亡率和婴幼儿死亡率；减少出生缺陷发生，提高出生人口素质。
　　人口计生委：保护妇女实行计划生育的权利；组织开展人口和计划生育宣传教育，倡导婚育新风，普及有关法律法规以及优生优育、避孕节育、生殖健康的科学知识；开展计划生育和生殖健康优质服务，保护育龄妇女身心健康；开展关爱女孩行动和救助贫困母亲的活动，营造有利于女孩及生育女孩母亲的良好生存环境；继续加强和完善基层计划生育网络建设，努力稳定低生育水平，提高出生人口素质。
　　工商总局：依法保护妇女从事经济活动的平等权利；依法监督流通领域商品（包括服务）质量，查处假冒伪劣等各类侵害消费者，尤其是妇女儿童合法权益的违法行为；依法对广告进行监督管理，保护妇女儿童作为消费者的合法权益。
　　质检总局：推动妇女儿童用品和食品的质量、安全规范的立法工作，制定国家标准、行政规章、安全技术规范和强制性标准；对涉及有关妇女儿童的标准化工作实施统一管理，建立健全严格的市场准入、检验检测和监督检查制度；打击假冒伪劣产品，提供有关质量监督、检验检疫方面的信息指导和咨询服务，保护妇女儿童权益。
　　广电总局：宣传党和国家关于妇女儿童发展的法律法规、方针政策和目标任务；宣传妇女儿童工作以及广大妇女在改革开放和现代化建设中作出的贡献；为妇女儿童提供优秀的广播影视作品，禁止在广播影视中对妇女儿童形象的污辱性宣传。
　　新闻出版总署：做好宣传自尊、自信、自立、自强的女性和德、智、体全面发展的少年儿童的图书、报刊、音像电子读物的出版工作，为提高妇女素质和促进少年儿童的健康成长出版更多优秀读物；制止歧视妇女形象的图书、报刊和音像电子读物出版，查禁淫秽色情及危害未成年人身心健康的出版物。
　　体育总局：贯彻落实《全民健身计划纲要》和《学生体质健康标准》，广泛开展适合妇女儿童的全民健身运动，增强妇女儿童体质。
　　统计局：负责《[中国妇女发展纲要](https://www.pkulaw.com/chl/37733dd7246afab4bdfb.html)》、《中国儿童发展纲要》的统计监测工作，建立妇女儿童状况监测体系，加强妇女儿童发展综合统计工作，收集有关妇女儿童的统计数据；建立和完善妇女儿童数据库，为妇女儿童事业发展和政府决策提供统计咨询服务。
　　林业局：加强林业知识的宣传，提高广大妇女儿童的绿化和生态文明意识；支持和鼓励妇女参加义务植树、造林绿化、林业生态建设（包括“三八绿色工程”）等活动，提供林业科技、政策和信息服务。
　　法制办：积极推动保护妇女儿童权益、促进妇女儿童发展的法律法规的出台；在起草、审查法律和行政法规草案中，注意保护妇女儿童权益、促进妇女儿童事业的发展。
　　全国总工会：贯彻男女平等的基本国策，参与有关政策法规的研究制定，依法维护女职工的合法权益和特殊利益；努力提高女职工队伍的整体素质，团结动员女职工为全面建设小康社会建功立业。
　　共青团中央：加强对中国少年先锋队工作的领导，健全少先队各级工作机构，加强少先队辅导员队伍建设；教育引导少年儿童提高思想道德和综合素质；服务少年儿童学习、生活、健康、安全、娱乐等多方面需求；依法维护少年儿童的合法权益，促进少年儿童的健康成长。
　　全国妇联：推动妇女积极参与全面建设小康社会事业，推动妇女参与社会主义经济建设、政治建设、文化建设和社会建设，促进妇女为经济社会发展作贡献；促进妇女全面提高自身素质，实现自身的进步与发展；参与贯彻实施《[中国妇女发展纲要](https://www.pkulaw.com/chl/37733dd7246afab4bdfb.html)》和《中国儿童发展纲要》；依法维护妇女儿童的合法权益；承担国务院妇女儿童工作委员会办公室的工作；促进国内外妇女之间的交往与合作；推动优化儿童健康成长的社会环境；促进妇女儿童事业的发展。
　　中国残联：依法维护残疾妇女和儿童的合法权益；参与和推动保护残疾妇女和儿童法律、法规的制定和完善；促进和提高残疾妇女儿童的康复、教育和发展水平。
　　中国科协：推动妇女儿童智力开发；组织妇女儿童科普教育活动和城乡妇女实用技术培训；促进妇女儿童科技文化素质提高和女科技工作者健康成长。
　　中国关工委：关心未成年人健康成长，积极参与和推动《中国儿童发展纲要》的实施和儿童合法权益保护工作；配合有关部门加强青少年思想道德建设，净化青少年成长环境；发挥“五老”（老战士、老干部、老教师、老模范、老专家）优势，以多种形式对青少年进行思想品德、理想信念、社会主义价值观和人生观教育；关心少年儿童特别是弱势群体的学习与生活，有计划地适当组织关爱活动；针对少年儿童成长中遇到的各类问题，组织开展调查研究并积极向有关部门反映情况、提出建议。

© 北大法宝：（[www.pkulaw.com](https://www.pkulaw.com/)) 专业提供法律信息、法学知识和法律软件领域各类解决方案。北大法宝为您提供丰富的参考资料，正式引用法规条文时请与标准文本核对。
欢迎查看所有[产品和服务](https://www.pkulaw.com/helps/2.html)。法宝快讯：[如何快速找到您需要的检索结果？](https://www.pkulaw.com/helps/70.html)[法宝](https://www.pkulaw.com/helps/69.html)V6有何新特色？

扫描二维码阅读原文

**原文链接：**<https://www.pkulaw.com/chl/6a8e840d56357accbdfb.html>
